# African Lions and Zoonotic Diseases: Implications for Commercial Lion Farms in South Africa

**DOI:** 10.3390/ani10091692

**Published:** 2020-09-18

**Authors:** Jennah Green, Catherine Jakins, Eyob Asfaw, Nicholas Bruschi, Abbie Parker, Louise de Waal, Neil D’Cruze

**Affiliations:** 1 World Animal Protection 222 Gray’s Inn Rd., London WC1X 8HB, UK; JennahGreen@worldanimalprotection.org (J.G.); EyobAsfaw@worldanimalprotection.org (E.A.); NicholasBruschi@worldanimalprotection.org (N.B.); AbbieParker@worldanimalprotection.org (A.P.); 2 Blood Lion NPC, P.O. Box 1548, Kloof 3640, South Africa; cathjakins@gmail.com (C.J.); louise@greengirlsinafrica.com (L.d.W.)

**Keywords:** zoonotic disease, *Panthera leo*, human health, biosecurity, wildlife farming, wildlife trade, disease transmission

## Abstract

**Simple Summary:**

In South Africa, thousands of African lions are bred on farms for commercial purposes, such as tourism, trophy hunting, and traditional medicine. Lions on farms often have direct contact with people, such as farm workers and tourists. Such close contact between wild animals and humans creates opportunities for the spread of zoonotic diseases (diseases that can be passed between animals and people). To help understand the health risks associated with lion farms, our study compiled a list of pathogens (bacteria, viruses, parasites, and fungi) known to affect African lions. We reviewed 148 scientific papers and identified a total of 63 pathogens recorded in both wild and captive lions, most of which were parasites (35, 56%), followed by viruses (17, 27%) and bacteria (11, 17%). This included pathogens that can be passed from lions to other animals and to humans. We also found a total of 83 diseases and clinical symptoms associated with these pathogens. Given that pathogens and their associated infectious diseases can cause harm to both animals and public health, we recommend that the lion farming industry in South Africa takes action to prevent and manage potential disease outbreaks.

**Abstract:**

African lions (*Panthera leo*) are bred in captivity on commercial farms across South Africa and often have close contact with farm staff, tourists, and other industry workers. As transmission of zoonotic diseases occurs through close proximity between wildlife and humans, these commercial captive breeding operations pose a potential risk to thousands of captive lions and to public health. An understanding of pathogens known to affect lions is needed to effectively assess the risk of disease emergence and transmission within the industry. Here, we conduct a systematic search of the academic literature, identifying 148 peer-reviewed studies, to summarize the range of pathogens and parasites known to affect African lions. A total of 63 pathogenic organisms were recorded, belonging to 35 genera across 30 taxonomic families. Over half were parasites (35, 56%), followed by viruses (17, 27%) and bacteria (11, 17%). A number of novel pathogens representing unidentified and undescribed species were also reported. Among the pathogenic inventory are species that can be transmitted from lions to other species, including humans. In addition, 83 clinical symptoms and diseases associated with these pathogens were identified. Given the risks posed by infectious diseases, this research highlights the potential public health risks associated with the captive breeding industry. We recommend that relevant authorities take imminent action to help prevent and manage the risks posed by zoonotic pathogens on lion farms.

## 1. Introduction

Zoonotic diseases are infectious diseases caused by pathogenic agents (including bacteria, parasites, fungi, viruses, and prions) that can be transmitted between vertebrate mammals and humans [1]. Outbreaks of zoonotic diseases can have widespread consequences for public health and are thought to cause two billion cases of human illness and over two million human deaths every year [2]. Disease outbreaks from wild animal sources periodically result in hundreds of billions of dollars of economic damage [3]. The most recent global health pandemic, coronavirus COVID-19, which was also thought to originate in a wild animal host [4], is likely to cost the global economy between 5–9 trillion USD [5].

The increasing rate of emerging infectious diseases is thought to be a result of human-induced changes in land use, extraction of natural resources, animal production systems, and the global wildlife trade [6,7]. Wildlife harbor a large and often unknown reservoir of infectious diseases [8] and zoonotic disease transmission to people occurs when wild animals are in close proximity with human activity [6]. Most recent global health pandemics [9], including COVID-19, are thought to have originated in wild animal hosts [4]. A range of solutions could be put in place to prevent future zoonotic epidemics (see Petrovan et al. [10]). However, it has been suggested that efforts to decrease contact between wild animals and humans could prove to be the most practical and cost-effective approach in reducing the global public health threat posed by zoonotic emerging infectious diseases [11]. 

Commercial use of wildlife, whether legal or illegal, puts humans in direct contact with a range of wild species [12]. Wildlife farms (herein referred to as facilities that breed non-domesticated species for commercial purposes) in particular can create opportunity for pathogen transmission between wild animals and their human caretakers because of regular or prolonged contact for husbandry purposes [13]. Furthermore, conditions often associated with wildlife farms, such as high concentrations of wild animals in the same enclosures, poor hygiene, and stress associated with captive conditions, can reduce resistance to pathogens and increase the risk for transmission of disease [14,15]. 

A diverse range of wild animal species are farmed around the world for a range of commercial purposes, for example as exotic pets (e.g., snake farms in West Africa [16]), traditional medicine (e.g., bear bile farms in China and South-East Asia [17]), leather (e.g., alligators farms in the USA [18]), or fur (e.g., mink and fox farms in Europe [19]). Cases of infectious disease emergence from pathogen transmission among farmed wildlife have been documented from across the taxonomic spectrum. For example, transmission of zoonotic tape worm *Armillifer armillatus* from snakes to a farm owner was reported in The Gambia [20], and rapid transmission of coronavirus COVID-19 occurred recently between mink and farm workers at a mink farm in the Netherlands [21].

African lions (*Panthera leo*) are bred and kept on commercial farms across South Africa. These lions are bred for a range of purposes that can involve direct contact with people, including interactive tourism experiences (e.g., paying international volunteers working with predators and day tourists involved in cub petting and walking activities), recreational hunting for ‘trophies’, and bone exports to Asia for use in traditional medicine products [22,23]. For example, lion bone and trophy exports require a number of ‘middle-men’ who are required to have direct contact with lions and/or handle their derivatives during transport, slaughter, and/or preparation. This relatively high level of direct contact between lions and people (or consumption of their parts and derivatives) provides ample opportunity for zoonotic exchange. 

A review of diseases present among lions in the Kruger Park during the 1970s provides an important insight into the variety of infectious diseases that can affect populations in the wild (e.g., trichinosis, filariasis, sarcoptic mange, pentastomiasis, echinococcosis, taeniasis, hepatozoonosis, anthrax, and babesiosis), including some that are considered to be either directly or indirectly transmissible to humans [24]. Likewise, scientific studies have reported the transmission of zoonotic infectious diseases between humans and captive lions. For example, in 2015, a zoo-housed lion cub presented with ‘dermatophytosis’, a disease caused by infection with pathogenic fungi *Epidermophyton*, *Microsporum*, or *Trichophyton*, was also contracted by a zookeeper caring for the lion as a result of continuous contact with the animal [25]. 

The number of lions bred on farms in South Africa has grown exponentially in the last two decades to a current captive population of up to 8500 individuals housed across more than 300 facilities [22]. The vast scale of these intensive breeding facilities further increases the number of people in close contact with lions and the opportunities for zoonotic disease transmission. Yet, to the authors’ knowledge, despite its value for informing efforts to prevent, monitor, and manage any associated zoonotic disease outbreaks, no attempt has yet been made to compile a list of pathogenic organisms associated with African lions from recent scientific studies. Consequently, this review of the academic literature published in the last ten years provides an initial baseline of pathogenic organisms and discusses the potential animal and public health risks associated with the captive predator industry. 

## 2. Materials and Methods 

We conducted a systematic review of the scientific literature using the academic journal database Web of Science (Philadelphia, PA, USA). A total of 13 search terms relating to pathogenic health were searched on the database (Disease, Pathogen, Virus, Viral, Bacteria, Bacterial, Parasite, Parasitic, Fungus, Fungal, Zoonosis, Zoonotic, and Health). Each search term was employed with the Boolean operator ‘AND’, with the additional term *Panthera leo*. Searches were conducted for the time period 2009–2019, which returned a total of 252 results, comprising 152 individual academic papers. Of the 152 papers returned from the literature search, one could not be sourced due to institutional access and three were excluded because they were not published in English. The remaining 148 papers are included in the analysis. 

Each paper was examined by one of six reviewers, who recorded any mention of ‘bacteria’, ‘fungi’, ‘parasite’, ‘protozoa’, or ‘virus’ in each article. All disorders, diseases, or conditions were recorded in relation to African and Asiatic lions, with a list of specific named pathogenic organisms compiled. The environment in which the lions were studied was recorded (wild or captive) with specific details on the type of captivity lions were housed in (commercial enterprises, zoos, private ownership, or mixed purposes). In addition, the papers were reviewed for information about disease transmission. The reviewers recorded where it was specified that pathogenic organisms were transmissible between African lions and other animal species, as well as between African lions and humans.

## 3. Results

A total of 63 different pathogenic organisms, known to affect lions, were reported (Table 1). Over half of the reported pathogenic organisms were parasites (35, 56%), including ticks (order Ixodida) (4, 6%), followed by viruses (17, 27%), and bacteria (11, 17%), with no pathogenic fungi reported. These 63 pathogenic organisms belong to 35 different genera across 30 different taxonomic families. Three novel pathogenic organisms representing unidentified and undescribed species were also reported. 

The review also identified a total of 83 clinical symptoms and diseases associated with these pathogenic organisms (Table 2), highlighting the range of detrimental health risks that these pose to their feline hosts. With regards to information on the transmission of infectious disease, 38 (26%) of the scientific papers referred to transmission between lions and other species and three (2%) specifically referred to transmission between lions and humans. 

Of the 109 papers that focused on African lions, 45 (41%) were based on data from captive lions, 61 (56%) from wild lions, and three (3%) from a mixture of both. Of the studies focusing on captive lions, only one collected data from a commercial breeding facility in South Africa. The study used samples from three deceased lions to analyze their evolutionary history and was unrelated to pathogens or disease. One further study focused on commercial facilities in South Africa but did not collect first-hand data and instead used literature sources to review the suitability of captive bred lions for reintroduction into the wild. The remainder of the captive data came from lions housed in zoos, wildlife sanctuaries, and reserves (34, 76%), in private ownership (5, 11%), or a combination of both (4, 9%).

## 4. Discussion

A systematic review of scientific literature confirmed that a range of 63 different pathogenic organisms are known to exist in both captive and wild free ranging lions (Table 1 and Table 2). A number of novel pathogenic organisms, in some cases representing unidentified and undescribed species, were reported, including novel *Babesia* species and *Cystoisospora*-resembling oocysts. 

There is a paucity of knowledge on disease susceptibility, transmission, epidemiology, and pathology in lions [100]. While the list of known pathogenic organisms will undoubtedly grow, this review provides an important baseline inventory. Given the conditions in which the lion farming industry currently operates, the considerable scale of trade in lions, and their susceptibility to such a wide range of multi-host pathogenic organisms, it is likely that farmed lions could play a central role in the emergence, amplification, and transmission of disease to both people and wild animal populations. 

### 4.1. Significance for Lion Health 

Some of the pathogenic organisms reported in this review are of significant health concern for captive and wild lions. For example, *Babesia* parasites, *Mycobacterium bovis* (a bacteria known for causing tuberculosis), canine distemper virus (CDV), canine parvovirus (CP), and feline panleukopenia virus (FPLV) (Table 1) are all highly contagious and cause significant morbidity and mortality in susceptible carnivore species. Infection with these pathogens is associated with a range of clinical symptoms, including but not limited to: emaciation, alopecia, diarrhea, seizures, recurrent twitching, and depression (Table 2) from which infected lions can suffer.

Some of these pathogenic organisms are particularly difficult to manage because infection of susceptible animals does not require direct physical contact. Infected lions shed pathogenic organisms in feces and other bodily secretions, e.g., aerosolized respiratory secretions [105,106] facilitating environmental contamination and rapid spread of disease. Furthermore, some of these organisms have longer incubation periods and can therefore lie undetected in the animals’ systems until they reach hazardous levels. For example, tuberculosis onset is slow, in many cases with the majority of infected lions initially appearing healthy [100,107]. In a captive setting, this renders detection and prevention of spread of infections between individuals housed together very difficult. 

In addition, some of these pathogenic organisms are likely to present a management challenge on commercial lion farms, as the onset of disease in lions often occurs suddenly after high stress situations, for example after repeated periods of pregnancy and lactation [100]. It has been suggested that intensive farming conditions and poor hygiene may be increasing the incidence of FPLV in captive carnivores, such as lions [108]. Disease transmission is promoted in immunocompromising conditions, and direct human–wildlife contact mixed with limited health and safety standards are all criteria for an emerging zoonosis hotspot [12]. 

Another challenge for captive facilities is that seemingly innocuous pathogens can cause harm when lions are ‘co-infected’ with multiple pathogens. For example, severe mortalities have occurred when individual lions were infected with both babesiosis and CDV, resulting in severe diseases like pneumonia and encephalitis, despite appearing healthy when infected with babesiosis alone [81]. This heightens the challenge of identifying infected individuals to manage diseases before transmission can occur. 

### 4.2. Significance for Human Health 

In addition to the potential significance for lion health, many of the pathogenic organisms reported in the reviewed scientific literature raise concerns for human health. For example, pathogenic strains of the Enterobacteriaceae *Escherichia coli* [26], the parasitic Sarcocystidae *Toxoplasma gondii* [14,87], and potentially, the parasitic Toxocaridae *Toxascaris leonine* [109] have a possible fecal–oral transmission route from lions to humans. For others, such as the Rickettsiaceae *Anaplasma phagocytophilum* [27], transmission via the bite of an infected arthropod tick is also possible. 

Some pathogens possess the capacity to infect human tissue using keratin and therefore only require physical contact with the lion’s fur; for example, *Microsporum gypseum*, the cause of dermatomycosis [110]. The adoption of prophylactic measures for sanitary maintenance for these animals and the professionals who maintain contact with them is paramount to reduce possible transmission of infection but is difficult to manage because of the asymptomatic nature of the pathogens in healthy lions [110]. Visitors to lion farms in South Africa have reported that basic hygiene protocols, for example hand sanitizing and stepping points to disinfect shoes between enclosures, are often absent for those intending to interact with the animals [111].

Lions have also been reported as hosts for diseases listed by the World Health Organization (WHO) as ‘neglected tropical diseases (NTDs)’ [112]. For example, human African trypanosomiasis caused by trypanosomes are multi-host parasites capable of infecting a wide range of wildlife species, including lions [93], that constitute a reservoir of infection for both people and domestic animals. *Echinococcosis*, a parasitic disease caused by tapeworms that reside in the intestines of carnivores, including lions [52], can cause serious morbidity and death in people. The prevalence of *Echinococcosis* is increasing in some African countries due to frequent contact between game animals (reservoir hosts), domestic animal hosts (such as dogs), and humans who are susceptible to transmission [113]. Neglecting these parasites can have severe socioeconomic consequences [113]. 

Captive carnivores can be predisposed to infections of *Toxoplasma*, a protozoan parasite with significant zoonotic potential [113]. Lions in particular have been identified as a susceptible host species [113]. Lions infected with *Toxoplasma* can transmit the parasites to people via blood and feces, causing severe pulmonary, cardiac, and brain inflammatory reactions (among others), sometimes with fatal outcomes [113]. Some *Toxoplasma* species have also been reported to cause abortion and fetal death; underestimating the impact of these parasites on humans could lead to a future epidemic where reduction in life expectancy, and increased child and maternal death, are rife [113].

Lions are also vulnerable to bovine tuberculosis (bTB), a disease caused by infection with the bacterial pathogen *M. bovis* [32,47]. Tuberculosis transmission at the wildlife–livestock–human interface is a growing concern worldwide, particularly in sub-Saharan Africa where infection is spreading [114]. Lions initially contracted bTB from infected buffalo carcasses [32], and although no direct spill-over from wildlife to humans (or vice-versa) has yet been documented [114], it is a growing concern, particularly in countries such as South Africa where there are a relatively high number of people living with HIV [115,116] and because HIV is the strongest known risk factor for TB [32]. Transmission of other pathogenic strains of tuberculosis from wild captive animals to humans has already been documented [117]. 

Epidemics caused by cats are possible (e.g., canine distemper in big cats) but are considered to be relatively rare [118]. While no evidence of lion-to-human transmission of feline coronavirus exists, isolation of pathogens with pandemic potential from any mammalian host is significant as it may provide conditions suitable for the virus to adapt to other mammalian hosts, enabling efficient mammal-to-human, and possibly also human-to-human, transmission, paving the way for a potentially devastating pandemic [119].

For example, it has recently been confirmed that big cats, including lions, can be infected with Sars-CoV-2 [120]. Some experts have publicly stated their belief that it is unlikely Sars-CoV-2 will naturally spread in a wild big cat population [118]. However, given the fact that the lions and tigers that tested positive for Sars-CoV-2 in the Bronx Zoo were likely to be infected by a zoo employee [121,122], there are on-going concerns that this virus could be passed from humans to big cats and vice versa in scenarios that involve captive individuals [118]. 

### 4.3. Significance for Lion Farming 

The maintenance of wild species in captivity provides an opportunity for unnatural human-wildlife proximity, facilitating interspecies sharing of pathogenic organisms [123]. Lions are kept in captivity in zoos in many cases as part of conservation breeding programs [124], but also, and in far greater numbers, on commercial wildlife farms [125]. While published data detailing the scale of wildlife farming and lion farming in particular in South Africa are scant, the South African Minister of Environment, Forestry and Fisheries stated in July 2019 in response to a Parliamentary question that the captive lion population in South Africa amounted to 7979 lions housed across 366 facilities.

**Figure 1:**
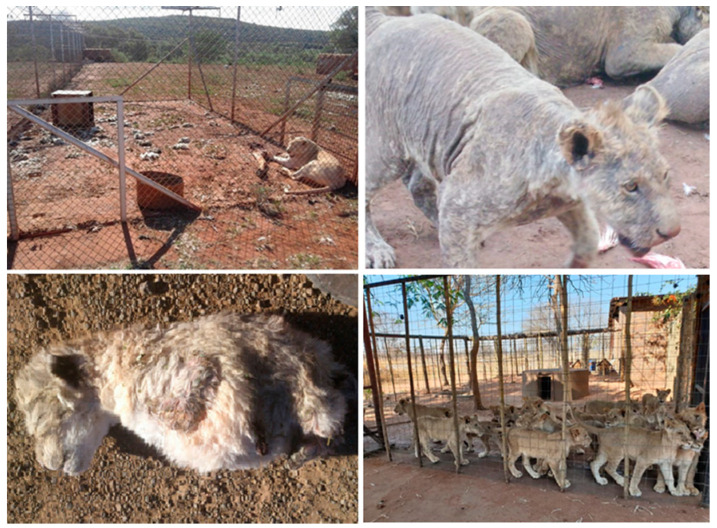
Environments provided for lions at commercial captive breeding facilities in South Africa are frequently reported as low welfare, involving large numbers of lions, often in poor physical condition and in over-crowded spaces. (**Top left**) Lioness housed in an enclosure with fecal matter and decaying carcasses. (**Top right**) Lions with little to no fur left as a result of severe and untreated mange. (**Bottom left**) Lion cub born with severe deformities, likely due to inbreeding. (**Bottom right**) Lions housed in overcrowded conditions. Images copyright Blood Lions.

Scientific papers that focus on the welfare conditions on commercial lion farms prevalent across South Africa are currently lacking. However, the living conditions and environments provided are frequently reported as low welfare, involving large numbers of lions, often in poor physical condition and in over-crowded spaces [126,127] (Figure 1). High concentrations of wild animals in the same enclosures can increase the risk for transmission of disease to and from wild animals due to reduced resistance to pathogens from factors associated with captivity, such as poor hygiene, poor diet, or stress [14,128]. Furthermore, cub separation from their mothers and the provision of alternative milk formulas (a practice reported at some lion farms [111]] can lead to nutritional deficiencies [129], which weakens immune systems and leaves animals more susceptible to pathogens [130].

A key part of this industry is “ecotourism”, where people are provided with the opportunity to come into close and unnatural proximity with lions via cub “petting” and “walking with” interactions or international volunteers paying to hand-rear lion cubs. The process of preparation of carcasses for human consumption also presents a considerable risk for transmission of disease to and from wild animals [131], a risk that is amplified in situations where slaughter and preparation take place at unregulated slaughterhouses, unbound by official hygiene standards [132]. Furthermore, the regulatory body that governs the international export of lion bones (The Convention of International Trade of Endangered Species, ‘CITES’) dictates quotas based on conservation science [133] and is not specifically aimed at preventing zoonotic disease introduction, despite the major role wildlife trade has as a transmission pathway for pathogenic organisms [12].

It is also important to note that any pathogenic organisms present in the captive lion population may pose a threat to the conservation of wild populations, particularly in scenarios where lion farms are located close to a wild lion habitat and where lion farm staff and visitors are actively engaged in other activities (e.g., conservation-focused field research, hunting, and photo tourism) that bring them into close proximity to free-ranging lions. For example, wild racoon dogs (*Nyctereutes procyonoides*) are thought to have transmitted CDV to a group of zoo-housed tigers in Japan [42] and records of transmission of intestinal nematodes between captive felids and local feral cats have been reported in Brazil [134]. Multi-host pathogenic organisms may pose a particular threat in scenarios where lion farm activity overlaps with areas inhabited by other free-ranging carnivore species (both wild and domesticated).

### 4.4. Mitigating Animal and Public Health Risks 

Remedial measures, such as improved animal welfare standards, veterinary interventions, and biosecurity protocols can partially mitigate the risk of zoonosis at captive lion breeding facilities [135]. However, due to the potential of asymptomatic pathogens affecting lions [110], biosecurity would require sophisticated disease surveillance, which could prove challenging [136,137]. Even with comprehensive surveillance, identification of emerging pathogens is still a challenge that poses significant animal and public health risks [12]. There is currently no publicly available information detailing the biosecurity protocols and regulatory standards within the lion breeding industry and, from our initial review of the literature, an apparent lack of national norms and standards for the health of the lions housed on commercial farms. 

Alternatively, a phased reduction in the scale of, or end to, the commercial captive breeding of lions for non-conservation purposes in South Africa could help to remove the animal and public health risks associated with this industry. However, efforts focused on improving animal husbandry, reducing consumer demand for lions (and their derivatives), increased enforcement effort, and the provision of economic incentives for farm staff would need to be considered to prevent any unintended consequences on lion welfare, conservation, and local livelihoods.

### 4.5. Limitations

We acknowledge that restricting our search to a ten-year time period and to one academic database will limit the number of relevant articles in our review. In addition, we recognize that additional onsite research is required to determine the incidence and prevalence of particular pathogenic organisms (in both captive and wild lion populations) and to help identify which infectious diseases are more likely to affect them under certain conditions. However, it was not our intention to provide a comprehensive overview of all pathogens affecting African lions or to provide specific statistics on their occurrence. Rather, the aim of our study was to create a baseline inventory of key pathogens and associated diseases and to describe the potential associated health concerns for both lions and people. Although our review may omit some relevant pathogens, we hope to demonstrate that, by only scratching the surface of this field, we identify a previously neglected area of consideration that will stimulate increased attention in future.

## 5. Conclusions

There are many socio-cultural, political, economic, and conservation factors that create a complex and nuanced debate around the commercial captive lion breeding industry in South Africa [133]. However, all economic, ethical, and environmental considerations aside, the data presented here indicate that the industry poses a potential risk to wild animal and public health. This initial literature review reveals a long and varied list of pathogenic organisms known to affect African lions, some of which can be transmitted to people. Given the range of pathogens identified, the growth of the industry over the last couple of decades, and the increasing number of people who have direct contact with live lions and/or their parts and derivatives, we recommend that a closer examination of the current policies and practices associated with commercial lion farming is required, particularly under a biosecurity lens. Furthermore, to properly safeguard lion and public health, it is paramount that the recommendations of any such examinations should be acted on with clear time-bound objectives relating to both implementation and enforcement. 

## Figures and Tables

**Table 1:** Pathogenic organisms (categorized into bacteria, parasites, and viruses) specified in the 148 papers in the dataset.

Pathogen Type	Family	Genus/Species	Source
Bacteria	Actinomycetaceae	*Actinomyces*	[26]
	Anaplasmataceae	*Ehrlichia canis*	[27]
	Bartonellaceae	*Bartonella koehlerae subsp. boulouisii*; *Bartonella henselae*	[28]
	Clostridiaceae	*Clostridia*	[26]
	Enterobacteriaceae	*Escherichia coli*	[26]
	Mycobacteriaceae	*Mycobacterium bovis*	[29,30,31,32,33,34,35,36,37,38]
	Mycoplasmataceae	*Mycoplasma haemominutum*	[39,40]
		*Mycoplasma Hemoplasma* spp.	[41]
	Rickettsiaceae	*Anaplasma phagocytophilum*	[27]
	Streptococcaceae	*Alpha-hemolytic streptococcus*	[26]
Viruses	Paramyxoviridae	*Morbillivirus* spp.	[42,43]
		*Canine distemper virus*	[32,37,42,44,45,46,47,48,49,50,51,52,53,54,55,56,57,58,59]
	Caliciviridae	*Feline calcivirus*	[44,47,51,56,60,61,62]
		*Sapovirus Norovirus*	[56]
	Herpesviridae	*Feline herpes virus*	[44,47,51,60,62]
	Retroviridae	*Feline immunodeficiency virus*	[32,38,47,51,52,57,60,62,63,64,65,66,67,68,69,70,71]
		*Feline lentivirus*	[72]
		*Feline leukemia virus*	[65,73,74]
		*Feline panleukopenia virus*	[44,51,61]
		*Gammaretrovirus*	[74]
	Parvoviridae	*feline panleukopenia virus*	[51]
		*Parvovirus*	[75]
	Coronaviridae	*Feline coronavirus*	[47,62]
	Picobirnaviridae	*Picobirnavirus*	[76]
	Reoviridae	*Mammalian orthoreovirus*	[77]
	Papillomaviridae	*Papillomavirus*	[78]
	Smacoviridae	*Smacovirus*	[79]
Parasites	Babesiidae	*Babesia canis*	[80,81]
		*Babesia felis*	[80,82]
		*Babesia lengau*	[80]
		*Babesia leo*	[27,80,82]
		*Babesia.* spp.	[32,51,82]
		*Babesia vogeli*	[27,80]
		*Novel babesia (similar to lengau)*	[80]
	Ixodidae	*Rhipicephalus simus*; *Rhipicephalus sulcatus*; *Rhipicephalus appendiculatus*	[51]
		*Rhipicentor nuttalli*	[51]
	Angiostrongylidae	*Aelurostrongylus abstrusus*	[83]
		*Aelurostrongylus* spp.	[83,84]
	Sarcocystidae	*Cystoisospora* spp.	[85]
		*Cystoisospora felis like oocysts*; *Cystoisospora rivolta like oocysts*	[86]
		*Sarcocystis* spp.	[84]
		*Toxoplasma gondii*	[87,88]
	Theileriidae	*Cytauxzoon manul*	[27]
		*Theileria parva*; *Theileria sinensis*	[27]
	Diphyllobothriidae	*Spirometra pretoriensis*	[89]
		*Spirometra ranarum*	[89,90]
		*Spirometra theileri*	[89,90,91]
		*Spirometra* spp.	[84,85,92]
	Trypanosomatidae	*Trypanosome b. rhodesiense*; *Trypanosome congolense*; *Trypanosome brucei s.l.*	[93]
	Taeniidae	*Taeniid cestodes*	[84]
		*Taeniid* spp.	[85]
	Toxocaridae	*Toxascaris leonine*	[94,95]
		*Toxocara cati*	[84]
	Trichinellidae	*Trichinella* spp.	[96]
	Hepatozoidae	*Hepatozoon canis*; *Hepatozoon felis*	[27,82]

**Table 2:** Associated diseases and clinical symptoms recorded in the 148 papers in the dataset.

Category	Terms from Source Papers
Diseases	Babesiosis [54]; bovine tuberculosis [29,30,32,33,34,37,47,52,62,64,71,97,98,99]; echinococcosis [52]; bilateral pulmonary disease [100]; encephalitis [42,43]; neurologic disease [43,50]; gingivitis [69]; gallbladder adenocarcinomas [26]; kidney disease [26]; biliary cystadenomas [26]; bacterial septicemia [26]; interstitial pneumonia [26,43]; necrotizing and neutrophilic hepatitis [26]; rabies [53]; pneumonia [94]
Clinical symptoms	Acute neurologic involvement [50]; anemia [32]; anisokaryosis [78]; anorexia [41,42,50]; ataxia [43,100]; bilateral submandibular swelling [100], cachexia [69]; congested lungs [26]; corneal opacity [100]; dehydration [26,32,69]; dehydration and hypertension due to kidney disease [101]; depletion of lymphoid organs [50]; depressed serum albumin [32]; profound depression or stupor [43]; dermal and/or mucocutaneous perioral masses [78]; diarrhea [94,95]; disorientation [43]; dyspnea [100]; elbow hygroma [33]; emaciation [33,35,100]; enlarged abdominal area [26]; facial and forelimb myoclonus (recurrent twitching) [43]; feline sarcoids [78]; fibropapilloma [78]; grand mal seizure [43]; hematologic derangements [64]; hyperglobulinemia [69]; immune depletion [70]; convulsions [102]; head tilt [102]; opisthotonos [102]; incoordination [102]; blindness [102]; hypoalbunemia [32]; monoytosis [32]; intraductular cholestasis [26]; ivermectin induced blindness [103]; lethargy [41]; leukopenia [75]; loss of coat condition [69]; lymphadenopathy [33,69]; lymphocytic depletion in lymph nodes and spleen [69]; lymphopenia [42]; hyperglobulinemia [32]; macroscopic abnormalities in the liver [26]; malnutrition [95]; mandibular swelling [33]; mange [33]; marked alopecia [100]; nasal discharge [42]; neutrophilic splenitis [26]; nodular polycystic lesion [26]; obstruction of the intestine [94]; papillomas [69]; peribiliary cysts [26]; polycystic liver [26]; pulmonary and bone lesions [35]; pyrexia [42]; severe seizures [104]; shoulder abscess [33]; tachypnoea [100]; vomiting [94,95]
